# The grand blueprint for porous materials

**DOI:** 10.1093/nsr/nwaf401

**Published:** 2025-09-22

**Authors:** Feng-Shou Xiao

**Affiliations:** Key Lab of Biomass Chemical Engineering of Ministry of Education, College of Chemical and Biological Engineering, Zhejiang University, China

Porous materials, including activated carbons, zeolites, metal–organic frameworks (MOFs) and covalent organic frameworks (COFs), have found firm applications in the field of energy and catalysis, adsorption and separation, radioactive elements recovery, and medical and biological engineering [1–3]. In 2023, the global market of zeolite products reached 67.5 billion dollars, with sorbents (65%) and catalysts (20%) as the main products. These practical application scenarios impose rigorous standards on porous materials. The pore size should be capable of being adjusted to allow for variable guest molecules, while the pore structure should provide unimpeded diffusion [4,5]. Conventional porous materials with single-level pore structure suffer from limited diffusion ability [6]. In contrast, hierarchically porous materials with multi-level pore structure have been developed to extend their application scenarios and to improve the diffusion ability [7]. However, it should be noted that enhancing diffusion ability alone does not invariably result in optimal efficiency. Catalytic processes composed of complex and multi-step procedures require multi-level diffusion behaviors [8]. In a multifunctional catalytic system, rapid diffusion over short distances may lead to undesirable interactions between two kinds of sites. Conversely, extended diffusion distances frequently give rise to side reactions [9]. The synchronization of catalytic processes and diffusion behaviors can result in optimized efficiency, which renders the design of diffusion behavior at the molecular level of paramount importance for the development of next-generation porous materials.

In a recent perspective article published in the *National Science Review* (NSR) entitled ‘Pore science and engineering: a new era of porous materials’, Prof. Li-Hua Chen, Prof. Ming-Yuan He and Prof. Bao-Lian Su provided a comprehensive overview of the historical development of porous materials and outlined a clear future direction for next-generation porous materials [10]. In this paper, to our knowledge for the first time, the very numerous porous materials have been classified, depending on the characteristics of pore structure. The authors firstly provide a concise overview of the significance and development history of synthetic porous materials, emphasizing the enrichment of pore topology, chemical composition and pore size of novel porous materials. Due to the uniformity of their pore size at a specific length scale, these materials are categorized as ‘Porous Materials 1.0’. The single-level pore structure of these materials has been found to be susceptible to a significant challenge of diffusion limitation, which results in low efficiency. The authors then summarize an elaborate and effective way to solve the diffusion limitation, that is, the introduction of hierarchical porous structure. Hierarchy represents a consequence of the evolution of life (Hierarchy Law of Life). Construction of a hierarchical pore system in a single crystal can endow porous materials with both improved mass diffusion ability and high structural stability. The authors have designated these hierarchical porous systems with characteristics of multi-levels, interconnectivity and regularity as ‘Porous Materials 2.0’. The development of porous materials with novel topologies and hierarchical pore systems have contributed to both the academic and industrial research communities, and remains a highly active area of research to the present day.

**Figure 1.**
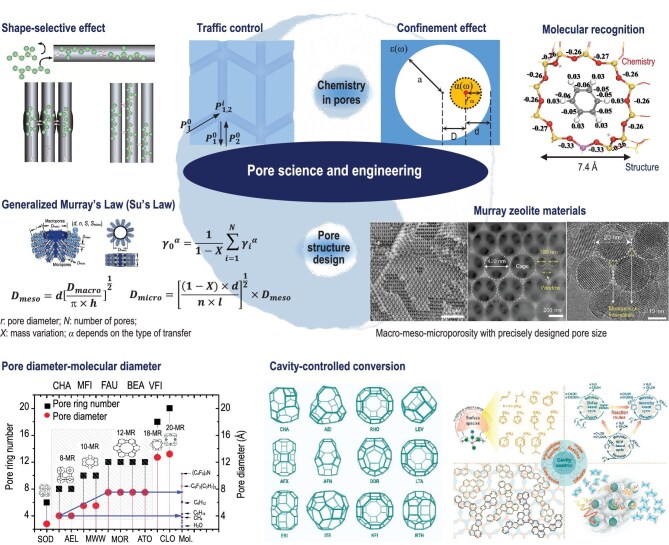
The connotation of pore science and engineering. Pore chemistry: shape-selective effect, traffic control, confinement effect and molecular recognition effect. Pore structure: generalized Murray’s Law, corresponding Murray materials and some relationships between pore structure and guest molecules in specific reactions. Adapted with permission from ref. [10]. Copyright from (2025) Oxford University Press.

For the first time, the authors have proposed a new discipline of ‘Pore Science and Engineering’ to gain further insights into diffusion behavior at the molecular level to rationally design pore structure and to establish a relationship between molecular behavior in the pore or a cage, and their diffusional and catalytic performance. Pore science and engineering comprises two major components: pore chemistry and pore structure (Fig. 1). Pore chemistry focuses on the molecular behavior in pores, encompassing such phenomena as shape-selective effect, traffic control, confinement effect and molecular recognition effect. These effects have been demonstrated to have a significant impact on molecular behaviors in pores. Pore structure design encompasses the generalized Murray’s Law, now named Su’s Law, for the quantitative relationship between the pore size at varying levels of the pore structure and the relationships between pore structure and guest molecules (reactants, intermediates and products) in specific reactions. These principles have contributed to the transformation of a research model from ‘trial-testing-modification’ to a more reactive approach of ‘reaction-demanded synthesis’ for the development of highly efficient porous materials, such as Murray materials (Fig. 1).

This pioneering discipline of ‘Pore Science and Engineering’ has given rise to novel concepts for understanding diffusion behavior at a molecular level and designing pore structure from a quantitative aspect. The authors emphasize the significance of comprehending molecule behavior in pores and the development of the theory of pore structure design to achieve diffusion improvement, with the ultimate objective being the precise design of diffusion behaviors. Due to the complexity and diversity of molecular behavior and pore structure, the authors call for devoted efforts from researchers and the aid of artificial intelligence (AI) to facilitate progress in this field. Under the guidance of ‘Pore Science and Engineering’, the next generation of porous materials are anticipated to exhibit meticulously designed and multi-level diffusion behaviors. With the assistance of AI, not only can the new porous materials be easily designed for a specific reaction, current porous materials in industrial processes as catalysts, adsorbents or others can also be optimized to exhibit enhanced efficiency and reduced consumption and emissions.
